# Association of physical activity with physical function and quality of life in people with hip and knee osteoarthritis: longitudinal analysis of a population-based cohort

**DOI:** 10.1186/s13075-023-02996-x

**Published:** 2023-01-26

**Authors:** David G. Lopes, Daniela Costa, Eduardo B. Cruz, Nuno Mendonça, Ana Rita Henriques, Jaime Branco, Helena Canhão, Ana M. Rodrigues

**Affiliations:** 1 EpiDoC Unit, NOVA Medical School | Faculdade de Ciências Médicas, NMS|FCM, Universidade Nova de Lisboa, Edifício Amarelo, Rua Do Instituto Bacteriológico, nº5, 1150-082 Lisbon, Portugal; 2 Comprehensive Health Research Centre (CHRC), NOVA Medical School | Faculdade de Ciências Médicas, NMS|FCM, Universidade Nova de Lisboa, Lisbon, Portugal; 3 NOVA National School of Public Health, Public Health Research Centre, Universidade Nova de Lisboa, Lisbon, Portugal; 4 Physiotherapy Department, School of Health, Polytechnic Institute of Setúbal, Setúbal, Portugal; 5 Centro Hospitalar Lisboa Ocidental (CHLO-E.P.E.), Serviço de Reumatologia Do Hospital Egas Moniz, Lisbon, Portugal; 6 Rheumatology Unit, Hospital Dos Lusíadas, Lisbon, Portugal

**Keywords:** Osteoarthritis, Health-related quality of life, Physical function, Exercise, Longitudinal

## Abstract

Hip and knee osteoarthritis (HKOA) is a chronic disease characterized by joint pain that leads to reduced physical function and health-related quality of life (HRQoL). At present, no cure is available. Clinical trials indicate that people with HKOA benefit from physical activity in several health-related outcomes. However, few studies have evaluated the long-term positive effect of regular physical activity. This study analyzed participants with HKOA from a nationwide population-based cohort (EpiDoC Cohort) to assess the impact of physical activity on patients’ physical function and HRQoL over a long-term follow-up. The regular weekly frequency of intentional physical activity was self-reported as non-frequent (0 times/week), frequent (1–2 times/week), or very frequent (≥ 3 times/week). This study followed 1086 participants over a mean period of 4.7 ± 3.4 years, during which 6.3% and 14.9% of participants reported frequent and very frequent physical activity, respectively. Using linear mixed models, we found that frequent (*β* =  − 0.101 [− 0.187, − 0.016]; *β* = 0.039 [− 0.002, 0.080]) and very frequent physical activity (*β* =  − 0.061 [− 0.118, − 0.004]; *β* = 0.057 [0.029, 0.084]) were associated with improved physical function and HRQoL over time, respectively, when compared with non-frequent exercise, adjusting for years to baseline, sex, age, years of education, body mass index, multimorbidity, hospitalizations, clinical severity, and unmanageable pain levels. These findings raise awareness of the importance of maintaining exercise/physical activity long term to optimize HRQoL and physical function. Further studies must address barriers and facilitators to improve the adoption of regular physical activity among citizens with HKOA.

**Supplementary Information:**

The online version contains supplementary material available at 10.1186/s13075-023-02996-x.

## Key messages

What is already known on this topic?

- Physical activity is potentially beneficial for physical function and HRQoL of people with HKOA.

What does this study add?

- Our results point to an improvement of physical function and HRQoL over a mean period of approximately 5 years in people with HKOA who reported frequent and very frequent physical activity relative to those who reported non-frequent physical activity.

How might this study affect research, practice, or policy?

- There is a need to address barriers and promote adherence to physical activity in the population with HKOA.
- Physical activity-based programs should be implemented in the early stages of HKOA and during the course of the disease as a core intervention for the management of HKOA and to prevent the clinical progression of the disease

## Introduction

Osteoarthritis (OA) is the most common type of arthritis and imposes a substantial burden on the individuals affected, including pain, disability, and markedly reduced quality of life [1]. It also poses a socioeconomic burden and considerable costs for healthcare systems [1]. OA is responsible for an estimated 9.6 million years lived with disability at the global level [2, 3]. The prevalence of hip and knee osteoarthritis (HKOA), the two most affected joints, has increased by 9.3% since 1990, and it is estimated that over 300 million people currently live with HKOA worldwide [2]. HKOA is a chronic disease characterized by joint pain and stiffness as well as limitation of movement that impairs health-related quality of life (HRQoL). At present, no cure is available [4]. Clinical trials indicate that exercise may improve physical function and HRQoL in people with HKOA [5, 6], and such studies have reported the beneficial effects of exercise programs on pain reduction and improvement of physical function and quality of life in people with HKOA [7, 8]. These findings have led clinical practice guidelines to recommend exercise and physical activity as core interventions for the management and prevention of HKOA [9]. However, most of the published literature has focused on the effects of exercise programs lasting up to 6–12 months; evidence of the long-term effects of regular exercise on the physical function and quality of life in people with HKOA is scarce.

Little epidemiological research has followed people with HKOA long term and analyzed the effect of exercise frequency on function and quality of life using longitudinal modeling. Analysis of variance (ANOVA) is commonly used in prospective studies wherein several measures are taken over time from the same participants. However, repeated ANOVA measures collapse observations across individuals or items, which leads to loss of information and thus statistical power. Multilevel modeling approaches, such as mixed-effects regression modeling, can overcome this problem [10]. In contrast to ANOVA, multilevel models are well adapted to handling variability within and across individuals, and they assume that missing observations are missing completely at random so as not to lose information.

Therefore, the present study aimed to assess the long-term impact of regular physical activity on physical function and quality of life among Portuguese citizens with HKOA using a prospective analytical approach.

## Methods

### Study design

This study used data from the Portuguese Epidemiology of Chronic Diseases (EpiDoC) cohort, a nationwide prospective cohort that enrolled a nationally representative random sample of non-institutionalized Portuguese adults (≥ 18 years old) between 2011 and 2013 [11]. There were four total waves of evaluation. The baseline evaluation (EpiDoC 1; 2011–2013) included 10,661 participants who were representative of the Portuguese adult population and was performed in two phases. First, a structured face-to-face interview was undertaken by a trained research assistant to screen for rheumatic and musculoskeletal diseases and collect sociodemographic and health-related data. In the second phase (*n* = 3877), a structured evaluation was conducted by a rheumatologist during a clinical appointment to validate the diagnosis of rheumatic and musculoskeletal diseases, as described elsewhere [3]. The three subsequent follow-up waves—EpiDoC 2, March 2013 to July 2015, *n* = 7591; EpiDoC 3, September 2015 to July 2016, *n* = 5653; EpiDoC 4, March to August 2021, *n* = 3757—were conducted using semi-structured phone call interviews in which a computer-assisted personal interview (CAPI) system delivered a core questionnaire, similar to that used in the first wave.

### Study population

This study included participants with a diagnosis of HKOA that was validated according to the American College of Rheumatology HKOA classification criteria, as described elsewhere [12]. The exclusion criteria were non-responses to the question, “Do you practice regular exercise/sports?,” or answering, “Doesn’t know/doesn’t answer,” and reporting a low physical activity frequency of less than once per week (rarely, sporadically, or occasionally).

### Outcomes assessment and definition

#### Physical function

Physical function was assessed in the four waves through the Health Assessment Questionnaire (HAQ), which evaluates physical limitations in daily activities through 20 questions with four levels each (without difficulty, some difficulty, with much difficulty, unable to do). A total score was computed for HAQ, ranging from 0 (no disabilities) to 3 (complete disability) [13].

#### Health-related quality of life

In the four waves, HRQoL was measured using the Portuguese validated version of the EQ-5D-3L questionnaire, which is composed of five dimensions (mobility, self-care, usual activities, pain/discomfort, and anxiety/depression) with three levels each (without problems, some problems, extreme problems). The descriptive system was converted into a summary index score ranging from 0 (equivalent to death; negative values correspond to states worse than death) to 1 (full health) [14].

### Exposure assessment and definition

#### Physical activity

Self-reported regular physical activity was assessed in each wave through the question, “Do you practice regular exercise/sports?,” with possible responses of “yes,” “no,” and “doesn’t know/doesn’t answer.” The frequency of intentional physical activity per week was defined as the number of days of exercise per week. Regular physical activity was defined as intentional exercise occurring at least once per week. Physical activity frequency was categorized into three subgroups: non-frequent (0 times per week), frequent (1 or 2 times per week), or very frequent (at least 3 times per week).

#### Covariates assessment and definition

Sex, age, nomenclature of territorial units for statistics II (NUTS II) region (Lisbon, North, Centre, Algarve, Alentejo, Madeira, and Azores), marital status, and education level were considered as sociodemographic variables. The NUTS II regions Madeira and Azores were considered as one Islands region. Marital status was categorized as “with partner” (married or consensual union) or “no partner” (single, widowed, or divorced). Education level was categorized as “ < 4 years” (less than primary education), “4–9 years” (primary or secondary education), or “ ≥ 10 years” (secondary or superior education). Body mass index (BMI) was categorized as “underweight/healthy weight” (≤ 24.99 kg/m^2^; combined due to the lack of representation in the underweight category), “overweight” (≥ 25 and ≤ 29.99 kg/m^2^), or “obese” (≥ 30 kg/m^2^), according to self-reported height and weight. Smoking habits (“never,” “in the past,” or “occasionally or daily”; the latter were combined due to few observations in the “occasionally” category) were also noted.

We defined multimorbidity as having ≥ 2 self-reported chronic non-communicable diseases of the following list of diseases considered at baseline: high blood pressure, high cholesterol, cardiac disease, diabetes mellitus, chronic lung disease, problems in the digestive tract, neurological disease, mental health disorders, allergies, cancer, and hyperuricemia. Self-reported hospitalizations in the previous year were coded as “yes/no.”

Clinical severity was evaluated at baseline with the Portuguese versions of the Knee Injury and Osteoarthritis Outcome Score (KOOS) [15] and the Hip Disability and Osteoarthritis Outcome Score (HOOS) [16]. A composite score encompassing the mean scores of each dimension of the assessments (pain, other symptoms, activities of daily living, sports and leisure, and quality of life) was computed and transformed into a 0–100 scale [17]. For easier interpretation, the inverted normalized mean score (0–100) was used, with higher values corresponding to higher clinical severity, as previously reported [18]. Pain intensity was measured as the mean pain intensity in the previous week with the 11-point Numeric Pain Rating Scale (NPRS) at baseline, and the population was divided into two subgroups: manageable pain levels (< 5 points) and unmanageable pain levels (≥ 5 points), according to the cutoff point for a manageable pain day in OA found by Zelman et al. [19]. In the case of both knee and hip being affected, the worse score of the two was considered.

Time-dependent variables were collected in all waves and included HRQoL, physical function, BMI, regular exercise, smoking habits, multimorbidity, and hospitalizations. The variables considered time-independent (only collected at baseline) were sex, NUTS II region, marital status, education level, disease severity, and unmanageable pain levels. The time in years since the baseline assessment was computed; therefore, only the age at baseline was considered to avoid multicollinearity.

### Statistical analysis

A descriptive analysis of the participants by their baseline frequency of physical activity was conducted using absolute (*n*) and relative frequencies (%) for categorical variables and mean ± standard deviation for continuous variables. Physical activity groups were compared using the chi-squared test (categorical variables) and Kruskal–Wallis test (continuous variables). Linear mixed models were used to assess the association of physical activity frequency (non-frequent, frequent, and very frequent) with physical function and HRQoL over time, considering varying intercepts for each participant and an independent covariance structure. Random slopes and other variance structures were tested but did not improve the models.

Univariate models were computed first to test the significance of potential predictors, with *p* < 0.25 as the selection criterion (Additional file 1: Table S1). The model-building process included comparing the models through likelihood ratio tests. Potential confounding variables were kept in the multivariate model if the literature supported their effects on physical function and HRQoL or if they achieved statistical significance of *p* < 0.05. Four models were built and adjusted for years from baseline. The interaction between physical activity and years from baseline was non-significant and thus not considered. NUTS II region, marital status, and smoking habits were also not included. Model 1 shows the crude effect of physical activity frequency. Model 2 was adjusted for sex, age at baseline, and education level. Model 3 was additionally adjusted for BMI. Model 4 was further adjusted for multimorbidity, hospitalizations, clinical severity, and unmanageable pain levels. The equation for the fully adjusted linear mixed model is:

$${y}_{ij}={\beta }_{00}+{\beta }_{1}{\mathrm{Physical \, activity}}_{ij}+{\beta }_{2}\mathrm{Year}{s}_{ij}+{\beta }_{3}{\mathrm{Sex}}_{1j}+{\beta }_{4}{\mathrm{Age}}_{1j}+{\beta }_{5}{\mathrm{Education}}_{1j}+{\beta }_{6}{\mathrm{BMI}}_{ij}+{\beta }_{7}{\mathrm{Multimorbidity}}_{ij}+{\beta }_{8}{\mathrm{Hospitalized}}_{ij}+{\beta }_{9}\mathrm{Clinical \, severit}{\mathrm{y}}_{1j}+{\beta }_{10}\mathrm{Unmanageable \, pain \, level}{\mathrm{s}}_{1j}+ {u}_{0j}+{\varepsilon }_{ij}$$

where physical function and HRQoL scores ($y_{ij}$) for observation $i$ in cluster $j$ depend on the overall mean intercept ${\beta }_{00}$, the coefficient ${\beta }_{p=1,\dots ,9}$ for the fixed effect ${x}_{ij}$, and the cluster-specific varying intercept ${u}_{0j}$. The overall error term is ${\varepsilon }_{ij}$. Fewer than 10% of the data were missing, so no imputation techniques were used. A sensitivity analysis was performed considering unmanageable and manageable pain levels subgroups, as well as clinical severity tercile subgroups (low, medium, and high). Analyses were carried out in STATA 17, and statistical significance was assumed at *p* < 0.05. Plots were derived using R version 4.1.1.

## Results

**Fig. 1:**
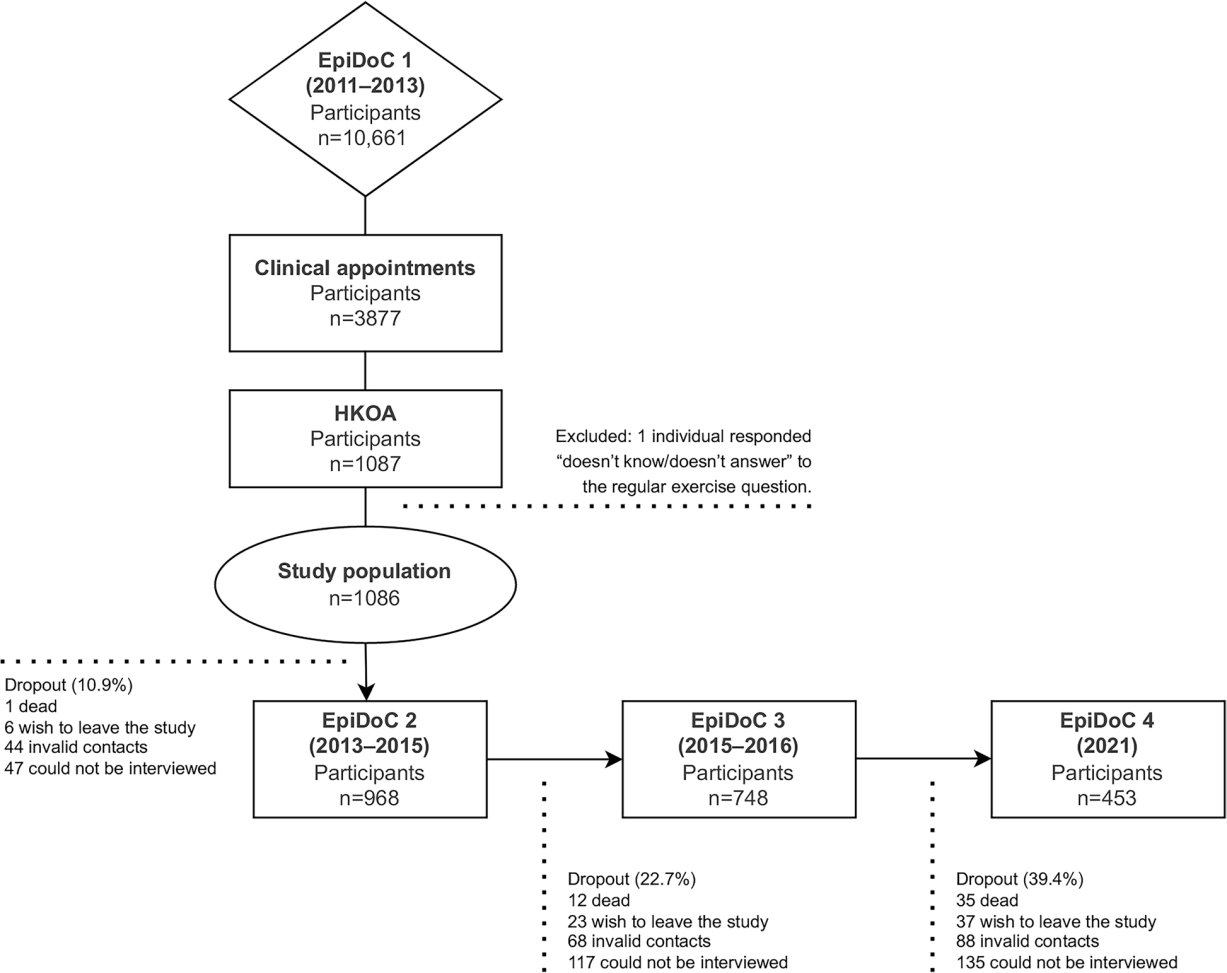
Flow diagram illustrating the participant eligibility and sample size for final analysis

This analysis included a sample of 1086 participants with HKOA who were followed over a mean period of 4.7 ± 3.4 years (Fig. 1).

The majority were women (*n* = 774, 71.3%). The cohort had a mean age of 65.4 ± 11.4 years, and most were overweight (*n* = 416, 41.3%) or obese (*n* = 400, 39.8%). Sixty-nine participants (6.3%) reported frequent physical activity and 162 (14.9%) very frequent physical activity. Univariate analysis showed that participants with non-frequent physical activity were older (mean = 66.1 ± 11.3; ≥ 75 years old: *n* = 213, 24.9%) and had a higher proportion of people with a lower education level (< 4 years of education: *n* = 240, 28.1%) than those with frequent or very frequent physical activity (*p* < 0.001). A higher proportion of the non-frequent physical activity subgroup reported multimorbidity (*n* = 566, 73.4%, *p* = 0.036) when compared with the other subgroups. There was also a higher proportion of people with unmanageable pain levels that reported non-frequent physical activity relative to the other subgroups (*n* = 618, 75.9%, *p* = 0.017) and a higher proportion of people with low clinical severity in the frequent (*n* = 25, 38.5%) and very frequent (*n* = 72, 49.7%) physical activity subgroups relative to non-frequent (*n* = 239, 30.4%, *p* < 0.001) (Table 1).

**Table 1:** Baseline sociodemographic, lifestyle, and clinical characteristics of HKOA participants according to the frequency of baseline physical activity, *n* (%)

	**All,** ***n* = 1086**	**Frequency of baseline physical activity**			***p***
		**Non-frequent, ** ***n* = 855**	**Frequent, ** ***n* = 69**	**Very frequent, ** ***n* = 162**	
**Sociodemographic**					
**Women**	774 (71.3%)	603 (70.5%)	57 (82.6%)	114 (70.4%)	0.099
**Age**					0.001
Mean (SD)	65.4 (11.4)	66.1 (11.3)	65.1 (9.7)	61.7 (12.2)	
< 55	187 (17.2%)	141 (16.5%)	8 (11.6%)	38 (23.5%)	
55–64	288 (26.5%)	211 (24.7%)	23 (33.3%)	54 (33.3%)	
65–74	368 (33.9%)	290 (33.9%)	27 (39.1%)	51 (31.5%)	
≥ 75	243 (22.4%)	213 (24.9%)	11 (15.9%)	19 (11.7%)	
**Region (NUTS II)**					0.502
North	290 (26.7%)	235 (27.5%)	17 (24.6%)	38 (23.5%)	
Center	268 (24.7%)	216 (25.3%)	18 (26.1%)	34 (21.0%)	
Lisbon	183 (16.9%)	134 (15.7%)	11 (15.9%)	38 (23.5%)	
Alentejo	74 (6.8%)	61 (7.1%)	3 (4.4%)	10 (6.2%)	
Algarve	22 (2.0%)	16 (1.9%)	3 (4.4%)	3 (1.9%)	
Islands	249 (22.9%)	193 (22.6%)	17 (24.6%)	39 (24.1%)	
**Marital status**					0.613
With partner	696 (64.1%)	544 (63.6%)	48 (69.6%)	104 (64.2%)	
**Education level**					< 0.001
< 4 years	268 (24.7%)	240 (28.1%)	8 (11.6%)	20 (12.5%)	
4–9 years	685 (63.1%)	543 (63.5%)	47 (68.1%)	95 (58.6%)	
≥ 10 years	133 (12.2%)	72 (8.4%)	14 (20.3%)	47 (29.0%)	
**Lifestyle**					
**BMI (kg/m**^**2**^**)**					0.119
Underweight/normal weight	190 (18.9%)	144 (18.4%)	10 (15.5%)	36 (22.9%)	
Overweight	416 (41.3%)	313 (40.0%)	34 (51.5%)	69 (44.0%)	
Obese	400 (39.8%)	326 (41.6%)	22 (33.3%)	52 (33.1%)	
**Smoking habits**					0.262
Never	812 (74.8%)	643 (75.3%)	55 (79.7%)	114 (70.4%)	
In the past	196 (18.1%)	145 (17.0%)	11 (15.9%)	40 (24.7%)	
Daily/occasionally	77 (7.1%)	66 (7.7%)	3 (4.4%)	8 (4.9%)	
**Clinical**					
**Multimorbidity**	704 (71.5%)	566 (73.4%)	39 (62.9%)	99 (65.1%)	0.036
**Hospitalization (previous year)**	130 (11.9%)	112 (13.1%)	6 (7.7%)	12 (7.4%)	0.084
**Unmanageable pain levels (≥ 5 NPRS)**					0.017
Yes	764 (73.9%)	618 (75.9%)	45 (66.2%)	101 (66.4%)	
**Clinical severity (inverted HOOS/KOOS)**					< 0.001
Low	336 (33.7%)	239 (30.4%)	25 (38.5%)	72 (49.7%)	
Medium	332 (33.3%)	266 (33.8%)	23 (35.4%)	43 (29.7%)	
High	328 (32.9%)	281 (35.8%)	17 (26.1%)	30 (20.7%)	

The sample size is not consistent due to missing values in some variables: BMI—all (*n* = 1006), non-frequent physical activity (*n* = 783), frequent (*n* = 66), very frequent (*n* = 157); smoking habits—all (*n* = 1085), non-frequent (*n* = 854); multimorbidity—all (*n* = 985), non-frequent (*n* = 771), frequent (*n* = 62), very frequent (*n* = 152); hospitalization—all (*n* = 1085), non-frequent (*n* = 854); unmanageable pain levels—all (*n* = 1034), non-frequent (*n* = 814), frequent (*n* = 68), very frequent (*n* =); clinical severity—all (*n* = 996), non-frequent (*n* = 786), frequent (*n* = 65), very frequent (*n* = 145)

The Islands region consists of Madeira and Azores. The chi-squared test notes NUTS II Alentejo and Algarve regions were merged due to an expected cell count < 5

*BMI*, body mass index; *NPRS*, Numeric Pain Rating Scale; *NUTS II*, nomenclature of territorial units for statistics II; *SD*, standard deviation

**Fig. 2:**
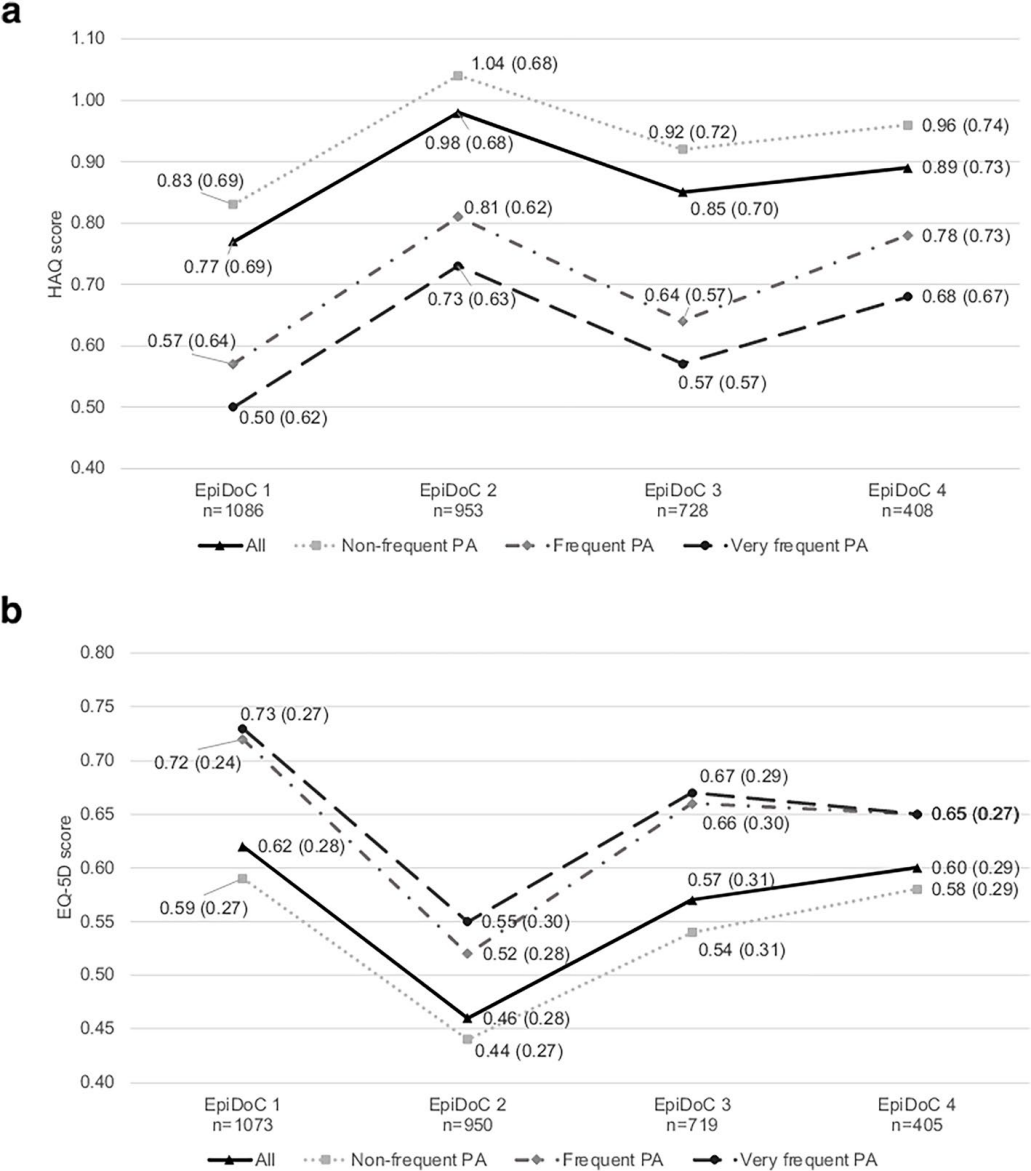
Average **a** physical function (HAQ score) and **b** HRQoL (EQ-5D score) of HKOA participants at baseline and in each follow-up wave (EpiDoC 2, 3, and 4). Data labels are mean (standard deviation). PA, physical activity. Sample size not consistent due to missing values: physical function EpiDoC2—all (*n* = 953), no (*n* = 751), frequent (*n* = 62), very frequent (*n* = 140); physical function EpiDoC3—all (*n* = 728), no (*n* = 567), frequent (*n* = 50), very frequent (*n* = 111); physical function EpiDoC4—all (*n* = 408), no (*n* = 297), frequent (*n* = 36), very frequent (*n* = 75); HRQoL EpiDoC1—all (*n* = 1073), no (*n* = 845), frequent (*n* = 67), very frequent (*n* = 161); HRQoL EpiDoC2—all (*n* = 959), no (*n* = 748), frequent (*n* = 61), very frequent (*n* = 141); HRQoL EpiDoC3—all (*n* = 719), no (*n* = 559), frequent (*n* = 50), very frequent (*n* = 110); HRQoL EpiDoC4—all (*n* = 405), no (*n* = 293), frequent (*n* = 37), very frequent (*n* = 75). Kruskal–Wallis test for the difference between physical activity categories: HAQ—EpiDoC 1 (*p* < 0.001), EpiDoC 2 (*p* < 0.001), EpiDoC 3 (*p* < 0.001), EpiDoC 4 (*p* = 0.005); EQ-5D—EpiDoC 1 (*p* < 0.001), EpiDoC 2 (*p* < 0.001), EpiDoC 3 (*p* < 0.001), EpiDoC 4 (*p* = 0.134)

The frequent and very frequent physical activity subgroups showed better HAQ scores (i.e., physical function) across the four waves, with all three groups differing significantly (*p* < 0.01) (Fig. 2a).

Similarly, the frequent and very frequent physical activity subgroups showed better EQ-5D scores across the four waves (Fig. 2b). There was a gradient in the EQ-5D scores according to physical activity frequency in the first three waves, with better scores in the frequent and very frequent subgroups (*p* < 0.001). In EpiDoC 4, there were no statistically significant differences between the subgroups.

**Fig. 3:**
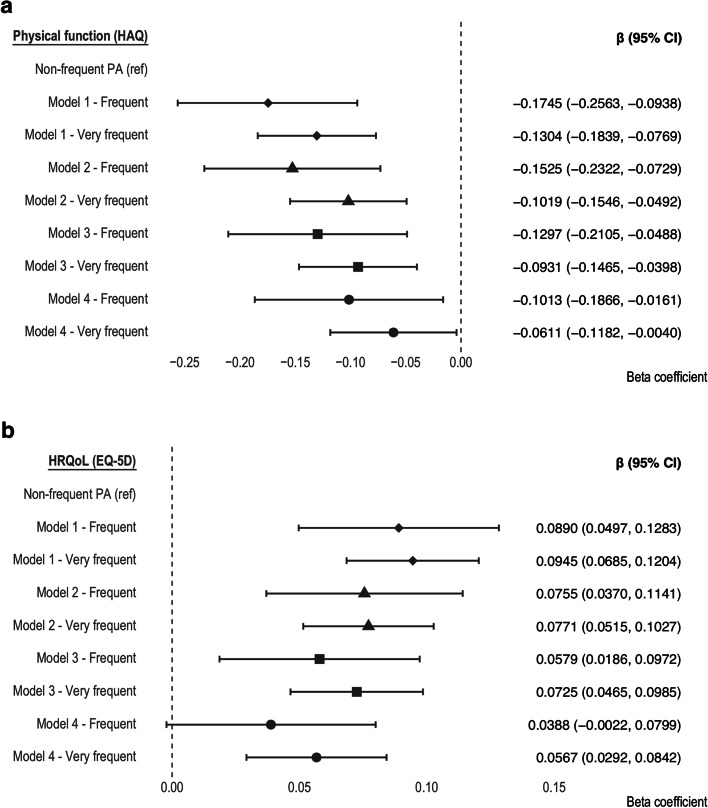
Estimates and 95% confidence intervals (95% CIs) of the association between physical activity frequency and **a** physical function and **b** HRQoL. PA, physical activity. Shapes indicate different models: diamonds for model 1, triangles for model 2, squares for model 3, and circles for model 4. All models are adjusted for years from baseline. Model 1 shows the crude effect of physical activity frequency. Model 2 was adjusted for sex, age group, and education level. Model 3 was further adjusted for body mass index. Model 4 was further adjusted for multimorbidity, hospitalizations, clinical severity, and unmanageable pain levels. Non-frequent physical activity was set as the reference in all models. Sample sizes (number of participants): physical function—model 1 (*n* = 1086), model 2 (*n* = 1086), model 3 (*n* = 1051), model 4 (*n* = 907); HRQoL—model 1 (*n* = 1084), model 2 (*n* = 1084), model 3 (*n* = 1050), model 4 (*n* = 907)

Using the non-frequent physical activity subgroup as a reference, we found a statistically significant association between the frequent/very frequent physical activity subgroups and the EQ-5D and HAQ scores over time in model 1, which adjusted only for years to baseline. This association remained independent from the progressive adjustments made for sex, age group, and education level in model 2; BMI in model 3; and multimorbidity, hospitalizations, clinical severity, and unmanageable pain levels in model 4, despite showing a decrease in the magnitude of the effects (Fig. 3).

Specifically, a negative association was found between physical function (HAQ score) and the frequency of physical activity; e.g., when adjusting for all confounding variables (model 4), the beta coefficients show that frequent (*β* =  − 0.101 [− 0.187, − 0.016], *p* = 0.020) and very frequent (*β* =  − 0.061 [− 0.118, − 0.004], *p* = 0.036) physical activity were associated with improvements in the physical function over time relative to non-frequent physical activity (Fig. 3a). Conversely, HRQoL (EQ-5D score) was positively associated with physical activity frequency; e.g., in model 4, frequent, though not significantly (*β* = 0.039 [− 0.002, 0.078], *p* = 0.064), and very frequent physical activity (*β* = 0.057 [0.029, 0.084], *p* < 0.001) were associated with improvements in HRQoL over time relative to non-frequent physical activity (Fig. 3b). Very frequent physical activity did not statistically differ from frequent physical activity despite showing a slightly higher effect estimate. Noteworthy, the reason why both the negative and positive associations found represent improvements is due to the scoring systems for HAQ and EQ-5D, where higher values are pessimistic in the former and optimistic in the latter, respectively.

Sensitivity analysis revealed frequent and very frequent physical activities were significantly associated with improvements in physical function and HRQoL in the subgroup with unmanageable pain levels. Very frequent physical activity was associated with improvements in HRQoL in the low clinical severity subgroup and improvements in the physical function and HRQoL in the high clinical severity subgroup. Although for the remaining subgroups the associations were not statistically significant, estimates pointed toward an improvement in both outcomes (Additional file 1: Table S2).

## Discussion

Using a community-based sample of Portuguese adults with HKOA, this study showed that regular physical activity was positively associated with long-term improvements in physical function and HRQoL. Model 4 shows results close to the statistical significance threshold. However, we recognize that statistical significance should not be placed above clinical significance [20]; the results should therefore be discussed in terms of the magnitude of effect [21]. Our results align with clinical trials that have shown that structured exercise programs improve short-term physical function and quality of life as well as several other outcomes, such as depression symptoms, self-efficacy, and social function [6, 7, 22]. Additionally, data from the Osteoarthritis Initiative also show that being physically active predicts performance in the 400-m walk test over a 4-year period [23]. The present study likewise shows that both frequent and very frequent physical activities are positively associated with long-term improvements in physical function.

The effects of different “doses” of physical activity or exercise on several osteoarthritis outcomes are contradictory in the literature. A systematic review by Regnaux et al. revealed that low-quality evidence showed no clinically important differences in pain and physical function between high- and low-intensity exercise programs [24]. However, in line with our results, a more recent systematic review by Kraus et al. showed that people who performed low levels of regular physical activity (at least 45 total minutes per week of moderate-intensity activity) saw improvements in physical function and HRQoL that were sustained over 6 months [5]. Previous literature has also suggested that the benefits of a physical activity program may be sustained for 6 months, after which the benefits decrease if regular physical activity is not maintained [8]. Therefore, maintenance of exercise and physical activity levels should be seen as a long-term goal in HKOA self-management interventions.

Our results show that a modest percentage of Portuguese citizens with HKOA regularly performed intentional physical activity ≥ 1 time per week (21.2%). This value is smaller than that in other cohorts [25] and the general Portuguese population [26]. Concerns regarding the low proportion of people who perform regular physical activity have also been raised in previous literature, which has pointed to a possible decrease of 14.4% in Portuguese people with high clinical severity of HKOA [12]. This reinforces the need for nationwide physical activity programs targeting people with HKOA.

In Portugal, there are no known national strategies or large-scale structured physical activity programs to promote physical activity among the OA population. “In general, core, non-pharmacological, conservative interventions such as exercise, healthy body weight management, or physiotherapy referrals seem to be infrequently implemented, and specialized care, e.g., orthopedic surgeon consultations, appears to be preferred.” [27]. Furthermore, OA is associated with an early exit from work in Portugal, defined as official early retirement or disability pensions in people < 65 years old, with annual indirect costs representing approximately 0.4% of the gross domestic product [28]. Creating opportunities for patients to maintain physical activity levels and exercise and addressing the barriers to doing so are crucial steps toward optimizing OA care outcomes and improving long-term adherence to physical activity as a self-management strategy [29] as well as decreasing the impact of HKOA at the patient and system levels. The results of this study emphasize regular and intentional physical activity as a modifiable factor that should be included in healthcare management programs for people with HKOA along with strategies that promote long-term adherence, such as health behavior change interventions.

The effects of physical activity frequency on physical function were not as obvious when the models were adjusted for all confounders. Hospitalizations, multimorbidity, clinical severity, and unmanageable pain levels may have decreased the association between physical activity frequency and physical function because these factors are also intrinsically related to decreased physical function [30]. Nevertheless, independent of the presence of these factors, there was a significant association that indicates that regular physical activity was more beneficial to maintaining higher levels of physical function in the long run than non-frequent physical activity.

This study has notable strengths. It used data from a nationwide study that prospectively evaluated a community-based sample of adults with a validated diagnosis of HKOA. The longitudinal approach and statistical analysis methods improved the robustness of the presented data and strengthened our conclusions regarding the predictive value of physical activity frequency on these two core outcomes of HKOA—physical function and HRQoL. However, some limitations should also be noted. We did not control for the type, duration, or intensity of physical activity sessions, which represent a gap previously highlighted in the literature. These important variables should be considered in future studies. Different types of physical activity— e.g., low-impact physical activity, competitions, and group sports—were also not taken into account in the analysis. We could also not distinguish between exercise (purposeful activity) and physical activity levels (any physical activity performed). Additionally, we could not conclude that these participants reached the international recommendations for weekly physical activity, and the comparison with other cohorts may be hindered. Moreover, the self-reported nature of the data, including physical activity frequency, hospitalizations, and chronic diseases, may potentiate recall bias.

## Conclusions

The findings of this study raise awareness on the importance of maintaining physical activity in people with HKOA to optimize physical function and HRQoL and calls for future research on the need to understand the barriers and develop strategies that can be effective in promoting long-term adherence to physical activity in the population with HKOA. Physical activity-based programs should be implemented early on as a core intervention for the management of HKOA and to prevent the clinical progression of the disease.

## Supplementary Information

**Additional file 1:** **Table S1.** Univariate linear mixed regression analysis of the factors associated with physical function and health-related quality of life (HRQoL). **Table S2.** Clinical severity and unmanageable pain level subgroup estimates and 95% confidence intervals (95% CIs) for the association between physical activity frequency and physical function and HRQoL.

## Data Availability

The codebook and analytical code are available from the authors upon request, and the dataset is available pending application approval by the EpiDoC coordinator, Ana Rodrigues (ana.m.rodrigues@nms.unl.pt).
