# Supplementary material for: Association of physical activity with physical function and quality of life in people with hip and knee osteoarthritis: longitudinal analysis of a population-based cohort
Source: Arthritis Res Ther. 2023 Jan 26;25:14. doi: 10.1186/s13075-023-02996-x (PMC9878813; doi:10.1186/s13075-023-02996-x)
Supplement: Supplementary file 1 — Additional file 1: Table S1. Univariate linear mixed regression analysis of the factors associated with physical function and health-related quality of life (HRQoL). Table S2. Clinical severity and unmanageable pain level subgroup estimates and 95% confidence intervals (95% CIs) for the association between physical activity frequency and physical function and HRQoL. [file 13075_2023_2996_MOESM1_ESM.docx]

**SUPPLEMENTARY MATERIAL**

**Table S1.** Univariate linear mixed regression analysis of the factors associated with physical function and health-related quality of life (HRQoL).

|  | $\boldsymbol{\beta}$ | **95% CI** |
| --- | --- | --- |
| **Physical function (HAQ)** |  |  |
| **Physical activity frequency** |  |  |
| No regular physical activity (ref) | - | - |
| Frequent | −0.185 | −0.267; −0.103 |
| Very frequent | −0.119 | −0.173; −0.064 |
| **Years to baseline** | 0.020 | 0.014; 0.026 |
| **Women** | 0.365 | 0.285; 0.443 |
| **Age group** |  |  |
| <55 (ref) | - | - |
| 55–64 | 0.165 | 0.058; 0.273 |
| 65–74 | 0.309 | 0.206; 0.412 |
| ≥75 | 0.587 | 0.475; 0.700 |
| **Region (NUTS II)** |  |  |
| North (ref) | - | - |
| Centre | −0.025 | −0.128; 0.077 |
| Lisbon | −0.124 | −0.238; −0.009 |
| Alentejo | 0.080 | −0.080; 0.274 |
| Algarve | −0.007 | −0.259; 0.274 |
| Islands | −0.073 | −0.178; 0.032 |
| **Marital status** |  |  |
| With partner | −0.125 | −0.202; −0.048 |
| **Education level** |  |  |
| <4 years (ref) | - | - |
| 4–9 years | −0.414 | −0.497; −0.332 |
| ≥10 years | −0.698 | −0.829; −0.578 |
| **BMI (kg/m^2^)** |  |  |
| Underweight/Normal weight (ref) | - | - |
| Overweight | −0.390 | −0.102; 0.025 |
| Obese | 0.104 | 0.030; 0.177 |
| **Smoking habits** |  |  |
| Never (ref) | - | - |
| In the past | −0.251 | −0.326; −0.177 |
| Daily/Occasionally | −0.261 | −0.388; −0.125 |
| **Multimorbidity** | 0.214 | 0.154; 0.273 |
| **Hospitalization**  **(previous year)** | 0.127 | 0.068; 0.185 |
| **Unmanageable pain levels (≥5 NPRS)** |  |  |
| Yes | 0.326 | 0.242; 0.409 |
| **Clinical severity**  **(inverted HOOS/KOOS)** |  |  |
| Low | - | - |
| Medium | 0.387 | 0.307; 0.466 |
| High | 0.763 | 0.684; 0.843 |
|  |  |  |
| **HRQoL (EQ-5D)** |  |  |
| **Physical activity frequency** |  |  |
| No regular physical activity (ref) | - | - |
| Frequent | 0.090 | 0.051; 0.129 |
| Very frequent | 0.930 | 0.671; 0.119 |
| **Years to baseline** | 0.001 | −0.003; 0.004 |
| **Women** | −0.123 | −0.153; −0.932 |
| **Age group** |  |  |
| <55 (ref) | - | - |
| 55–64 | −0.046 | −0.087; −0.005 |
| 65–74 | −0.090 | −0.129; −0.050 |
| ≥75 | −0.171 | −0.215; −0.128 |
| **Region (NUTS II)** |  |  |
| North | - | - |
| Centre | 0.016 | −0.023; 0.055 |
| Lisbon | 0.036 | −0.007; 0.080 |
| Alentejo | 0.005 | −0.056; 0.065 |
| Algarve | 0.023 | −0.077; 0.124 |
| Islands | 0.017 | −0.022; 0.057 |
| **Marital status** |  |  |
| With partner | 0.047 | 0.018; 0.076 |
| **Education level** |  |  |
| <4 years | - | - |
| 4–9 years | 0.148 | 0.116; 0.179 |
| ≥10 years | 0.252 | 0.206; 0.298 |
| **BMI (kg/m^2^)** |  |  |
| Underweight/Normal weight | - | - |
| Overweight | 0.006 | −0.024; 0.036 |
| Obese | −0.052 | −0.085; −0.019 |
| **Smoking habits** |  |  |
| Never | - | - |
| In the past | 0.913 | 0.060; 0.123 |
| Daily/Occasionally | 0.050 | −0.002; 0.102 |
| **Multimorbidity** | −0.105 | −0.132; −0.079 |
| **Hospitalization**  **(previous year)** | −0.057 | −0.086; −0.028 |
| **Unmanageable pain levels (≥5 NPRS)** |  |  |
| Yes | −0.143 | −0.175; −0.112 |
| **Clinical severity**  **(inverted HOOS/KOOS)** |  |  |
| Low | - | - |
| Medium | −0.148 | −0.179; −0.118 |
| High | −0.276 | −0.307; −0.246 |

$\beta$, beta coefficient; 95% CI, 95% confidence interval; ref, reference class, NUTS II, nomenclature of territorial units for statistics II; BMI, body mass index; NPRS: Numeric Pain Rating Scale.

**Sensitivity analysis**

**Table S2.** Clinical severity and unmanageable pain level subgroup estimates and 95% confidence intervals (95% CIs) for the association between physical activity frequency and physical function and HRQoL.

|  | **Physical function** | | | **HRQoL** | | |
| --- | --- | --- | --- | --- | --- | --- |
|  | $\boldsymbol{\beta}$ | **95% CI** | **n** | $\boldsymbol{\beta}$ | **95% CI** | **n** |
| ***Manageable Pain level*** | | | | | | |
| Exercise |  |  |  |  |  |  |
| Non-frequent | - | - |  | - | - |  |
| Frequent | −0.085 | −0.234; 0.063 | 248 | 0.009 | −0.065; 0.083 | 234 |
| Very frequent | −0.075 | −0.179; 0.028 |  | 0.015 | −0.037; 0.068 |  |
| ***Unmanageable Pain level*** | | | | | | |
| Exercise |  |  |  |  |  |  |
| Non-frequent | - | - |  | - | - |  |
| Frequent | −0.107 | −0.211; −0.003 | 699 | 0.052 | 0.003; 0.101 | 673 |
| Very frequent | −0.071 | −0.139; −0.002 |  | 0.074 | 0.041; 0.106 |  |
| ***Clinical severity - Low*** | | | | | | |
| Exercise |  |  |  |  |  |  |
| Non-frequent | - | - |  | - | - |  |
| Frequent | −0.101 | −0.215; 0.012 | 324 | 0.029 | -0.032; 0.090 | 320 |
| Very frequent | −0.016 | −0.089; 0.056 |  | 0.049 | 0.010; 0.089 |  |
| ***Clinical severity - Medium*** | | | | | | |
| Exercise |  |  |  |  |  |  |
| Non-frequent | - | - |  | - | - |  |
| Frequent | −0.124 | −0.267; 0.018 | 309 | 0.042 | −0.024; 0.109 | 308 |
| Very frequent | −0.025 | −0.131; 0.081 |  | 0.032 | −0.017; 0.080 |  |
| ***Clinical severity - High*** | | | | | | |
| Exercise |  |  |  |  |  |  |
| Non-frequent | - | - |  | - | - |  |
| Frequent | −0.076 | −0.286; 0.135 | 280 | 0.051 | −0.044; 0.145 | 279 |
| Very frequent | −0.201 | −0.332; −0.071 |  | 0.112 | 0.055; 0.170 |  |
|  |  |  |  |  |  |  |

Models adjusted for years from baseline, sex, age group, education level, body mass index, multimorbidity and hospitalizations. For pain management subgroups models were further adjusted for clinical severity, and for clinical severity subgroups models were adjusted for unmanageable pain levels. Non-frequent physical activity was set as the reference in all models.
